# The Year in Pediatric Electrophysiology: 2023

**DOI:** 10.19102/icrm.2024.15016

**Published:** 2024-01-15

**Authors:** Dustin B. Nash, Kathryn K. Collins

**Affiliations:** 1 Children’s Hospital Colorado, University of Colorado School of Medicine, Aurora, CO, USA

**Keywords:** Atrial standstill, catheter ablation, congenital heart disease, leadless pacing

## Introduction

This year in review commentary on pediatric electrophysiology reviews the most relevant studies of arrhythmias and their treatment in pediatric patients and those with congenital heart disease. The past year has seen knowledge advancements in catheter ablation, cardiac implantable electronic devices, and risk assessment and management of arrhythmias and associated conditions.

## Arrhythmias and associated conditions: risk assessment and management

Atrial standstill (AS) is a rare but described entity among pediatric patients characterized by the absence of electrical activity within the atria. A multi-institution collaborative effort led by Howard et al.^1^ of 20 patients <18 years of age at the time of diagnosis of AS is the largest description to date of such patients. The median age at diagnosis was 6.6 years and was associated with a high frequency of both atrial (80%) and ventricular (40%) arrhythmias. Genetic testing demonstrated a high frequency of *SCN5A* variants (65%) typically associated with loss of function. Eighteen of 20 (90%) patients underwent pacemaker implantation.

Accelerated junctional rhythm (AJR) and junctional ectopic tachycardia (JET) are common postoperative arrhythmias following surgery for congenital heart disease. To identify candidate patients for preoperative/intraoperative treatment, Dasgupta et al.^2^ leveraged the high volume of surgical procedures at Boston Children’s hospital over 7 years to develop a risk-prediction score of postoperative AJR/JET. Of 6354 surgeries, AJR occurred in 215 (3.4%) and JET occurred in 59 (0.9%). Independent positive predictors of AJR/JET included younger age, heterotaxy syndrome, aortic cross-clamp time, ventricular septal defect closures, and atrioventricular canal repair. The risk-prediction score had a C-index of 0.72 (0.70–0.75), suggesting a moderate predictive value in the identification of patients with postoperative junctional rhythms.

Ventricular fibromas in children are a known risk factor for ventricular arrhythmias. Surgical resection is considered a primary therapy, although the risk modification of such therapy is unknown. El Assaad et al.^3^ described a 46-patient cohort who had undergone resection of fibromas following cardiac arrest, significant ventricular arrhythmias, or hemodynamic abnormalities, with a primary outcome of recurrent ventricular tachycardia (VT)/ventricular fibrillation (VF). Surgical resection significantly reduced the risk of life-threatening arrhythmias, with 45 (98%) being free of clinical VT/VF.

## Cardiovascular implantable electronic devices

The results of a multicenter collaborative study of the use of transcatheter leadless pacing in children were published by Shah et al.^4^ Among 15 centers, 62 of 63 (98%) patients had successful implantations at a mean age of 15 (±4.1) years, including 20 (32%) with congenital heart disease. Eight patients were younger than 9 years of age, and eight (12.6%) were implanted from the internal jugular vein. Complications were noted in 10 (16%) patients, including cardiac perforation/pericardial effusion, femoral venous thrombus, and one device retrieval and replacement. There were no deaths, infections, or device embolizations.

A creative use of a modified leadless pacemaker was published by Berul et al.^5^ In this series, a modified Micra pacemaker (Medtronic, Minneapolis, MN, USA) was incorporated into a header to allow connection with an epicardial lead for specific application in neonatal patients. This drastically reduced the size of the pulse generator to minimize the risk of skin erosion or other complications. The weight of the described patients was 1.35–2.68 kg, and all implantations were successful.

## Catheter ablation

Przybylski et al.^6^ relayed the results of an effort to describe the underlying conditions and characteristics of patients with hypertrophic cardiomyopathy and accessory pathways (APs). Among 345 patients with hypertrophic cardiomyopathy, 28 (8%) had ventricular pre-excitation. This was true among patients with isolated hypertrophic obstructive cardiomyopathy (HOCM) and those with storage disorders, metabolic disease, and genetic syndromes. A notable 21% of these patients had clinical atrial fibrillation. Among the 22 patients who underwent an invasive electrophysiology study, 23 true APs were discovered in addition to 16 fasciculoventricular fibers. Ablation was acutely successful in 13 of 14 patients in whom it was attempted, and one procedure was complicated by complete heart block after ablation of a high-risk midseptal AP. The authors concluded that these distinct demographics should be considered in the evaluation of patients with HOCM and APs.

Also reported was the experience of Zhang et al.^7^ in the treatment of ventricular pre-excitation–induced dilated cardiomyopathy in infants. Among these young patients with a mean age of 6 months and a weight of 8 kg, the mean left ventricular ejection fraction (LVEF) was 32.6%. All APs were located along the right lateral wall, and all APs were successfully ablated. The LVEF normalized in all but one case, although it seemed to take longer for more severe dysfunction to do so.
